# Using UV–Vis Titration to Elucidate Novel Epigallocatechin Gallate (EGCG)-Induced Binding of the c-MYC G-Quadruplex

**DOI:** 10.3390/ph18050719

**Published:** 2025-05-14

**Authors:** Justin Tang

**Affiliations:** Department of Biomedical Science, University of Guelph, Guelph, ON N1G 2W1, Canada; jtang17@uoguelph.ca

**Keywords:** G-quadruplex, c-MYC, Epigallocatechin gallate (EGCG), polyphenol, DNA binding, UV–Vis spectroscopy, cancer therapy, brain tumour

## Abstract

**Background/Objectives:** Aberrant expression of c-MYC drives aggressive cancers. A guanine-rich promoter sequence (Pu27) folds into a transcriptionally repressive G-quadruplex (G4). Epigallocatechin gallate (EGCG), the main green tea polyphenol, displays anticancer activity, but clear, easily replicated evidence for direct binding to the c-MYC G4 is lacking. We therefore obtained the first biophysical confirmation of an EGCG–c-MYC G4 interaction using routine UV–visible spectroscopy. **Methods**: A pre-annealed Pu27 G4 (5 µM) in potassium-rich buffer was titrated with freshly prepared EGCG (0–20 µM) at 25 °C. Full-range UV–Vis spectra (220–400 nm) were recorded after each addition, and absorbance variations at the DNA (260 nm) and ligand (275 nm) maxima were quantified across three independent replicates. **Results**: EGCG induced pronounced, concentration-dependent hyperchromicity at 260 nm, reaching ~8–10% above baseline at a 4:1 ligand/DNA ratio and exhibiting saturable binding behaviour. Concurrently, the 275 nm band displayed relative hypochromicity coupled with a subtle bathochromic shift. These reciprocal perturbations—absent in buffer-only controls—constitute definitive evidence of a specific EGCG•G4 complex most consistent with external π-stacking or groove engagement rather than intercalation. **Conclusions**: This study delivers the first rigorous, quantitative UV–Vis confirmation that a readily consumed dietary polyphenol directly targets the c-MYC promoter G4. By marrying conceptual elegance with methodological accessibility, it provides a compelling molecular rationale for EGCG’s anti-oncogenic repertoire, inaugurates an expedient platform for screening G4-reactive nutraceuticals, and paves the way for structural and cellular investigations en route to next-generation c-MYC-directed therapies.

## 1. Introduction

Aberrant gene expression stands as a fundamental hallmark of cancer development and progression. Oncogenes, genes whose altered expression or function contributes to the cancerous phenotype, such as the transcription factor c-MYC, play crucial roles in orchestrating vital cellular processes including cell cycle progression, proliferation, metabolism, and the inhibition of apoptosis. Consequently, the overexpression or deregulation of c-MYC is a frequent observation across a broad spectrum of human malignancies, including particularly challenging high-grade brain tumours like glioblastoma, where it correlates with poor prognosis [1,2]. Therefore, strategies aimed at targeting oncogene expression or function remain a central and critical goal in contemporary cancer drug discovery efforts.

The discovery and characterization of non-canonical DNA secondary structures, particularly G-quadruplexes (G4s), have unveiled novel and promising avenues for therapeutic intervention against cancer [3]. G4s are distinctive four-stranded structures formed within guanine-rich nucleic acid sequences. Their architecture relies on Hoogsteen hydrogen bonding between guanines within a plane to form G-quartets, which then stack upon one another. These structures are crucially stabilized by the presence of monovalent cations (typically K^+^ or Na^+^) coordinated within the central channel formed by the stacked G-quartets [4]. Intriguingly, bioinformatic analyses and experimental studies have revealed that sequences capable of forming G4 structures are significantly enriched in functionally important genomic regions, including telomeric ends and the promoter regions of numerous key genes, including a significant number of oncogenes like c-MYC, VEGF, and KRAS [5,6].

Specifically, the G4 structure located within the nuclease-hypersensitive element III_1_ (NHE III_1_) upstream of the P1 promoter in the human c-MYC gene is one of the most extensively studied promoter G4s. Its formation has been directly implicated in the regulation of c-MYC transcription, potentially acting as a transcriptional silencer element [7,8]. Structurally, these activities arise from the conjugated aromatic system and multiple phenolic hydroxyls that govern redox behaviour and metal-chelating capacity. Significantly, the unique four-stranded structure of the c-MYC G4 differs from standard duplex DNA, making it a potential therapeutic target. It has been shown that certain small molecules can bind to and stabilize this G4 structure. This stabilization can physically impede the cellular machinery required for c-MYC gene expression, effectively reducing c-MYC levels and hindering cancer cell proliferation [7,8,9,10]. This validates the c-MYC G4 as a promising target for anticancer drug development.

Natural products, particularly polyphenols derived from dietary and medicinal plants, have long been recognized for their diverse health-promoting benefits, encompassing antioxidant, anti-inflammatory and, notably, anticancer properties [11,12,13]. Epigallocatechin gallate (EGCG) stands out as the most abundant and arguably most bioactive catechin found in green tea (Camellia sinensis). It has been the subject of extensive research investigating its potential anti-tumour activities across various cancer models [12]. While many studies have focused on mechanisms involving enzyme inhibition (e.g., targeting kinases or epigenetic enzymes) and modulation of critical cell signalling pathways, the possibility of direct interactions with nucleic acids has also been proposed as a contributing factor to EGCG’s bioactivity [13]. Indeed, some studies, often employing more complex biophysical techniques (like Surface Plasmon Resonance or Fluorescence Resonance Energy Transfer) or focusing primarily on downstream cellular effects, have hinted at the capability of EGCG and related catechin-type polyphenols (e.g., catechin, epicatechin, epigallocatechin) to interact with DNA, including potential recognition of G4 structures [14,15]. However, direct biophysical evidence of binding using fundamental and widely accessible techniques like UV–Vis spectroscopy, specifically focusing on the well-defined c-MYC promoter G4 sequence (Pu27), is sometimes less emphasized or reported within broader studies [12,13,14,15].

**Figure 1:**
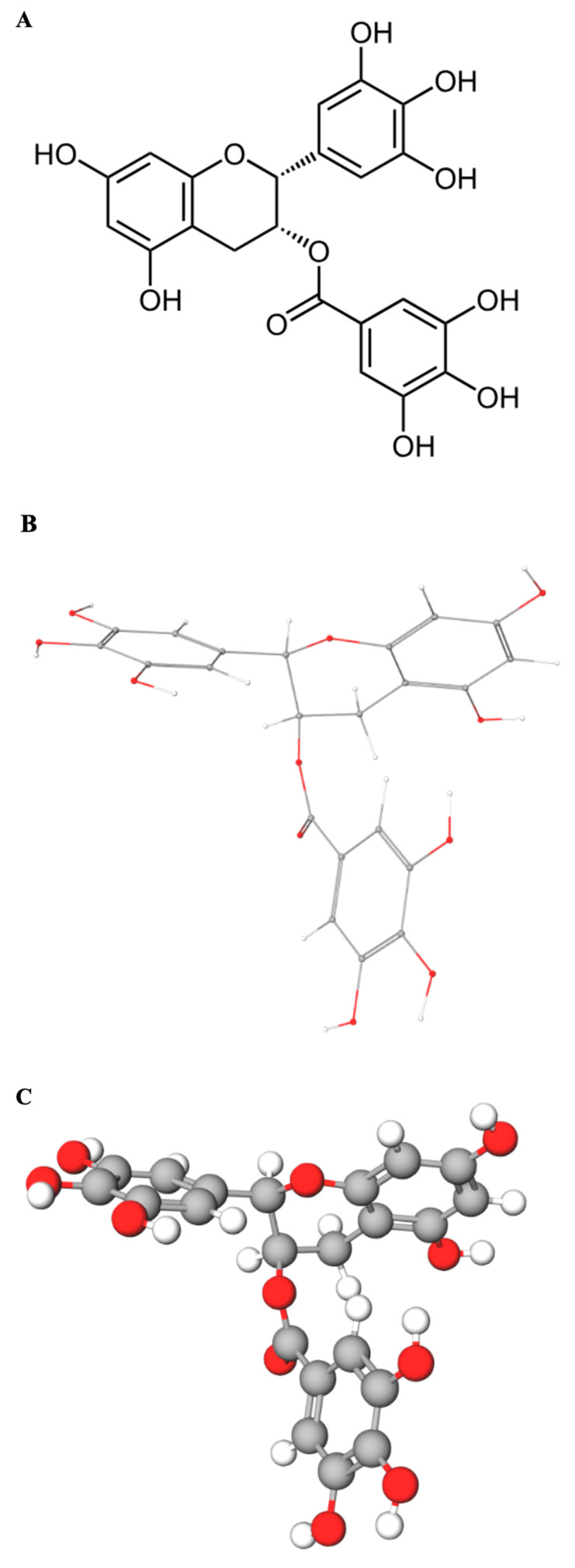
**Molecular representations of Epigallocatechin gallate (EGCG)**: (**A**) Two-dimensional line drawing of the flavan-3-ol core with a gallate ester at C-3. (**B**) Energy-minimized stick model (carbon, grey; oxygen, red; hydrogens in white) depicting the three aromatic rings and their relative orientation in three-dimensional space. (**C**) Space-filling (ball and stick) model highlighting the steric bulk and surface distribution of the eight phenolic-hydroxyl groups that confer EGCG’s high hydrogen-bonding and antioxidant potential.

UV–Vis absorbance spectroscopy represents a straightforward, cost-effective, and readily accessible technique ideally suited for monitoring interactions between small molecules (ligands) and macromolecules like DNA in vitro. The underlying principle is that binding events can perturb the electronic environment of either the DNA bases (chromophores absorbing around 260 nm) or the ligand chromophore (EGCG whose multiple phenolic rings contain conjugated π-electron systems that act as the primary chromophore, leading to strong UV absorbance with a maximum near 275 nm) (Figure 1A–C), or both.

Such perturbations typically manifest as measurable changes in absorbance intensity (hyperchromicity—increased absorbance, or hypochromicity—decreased absorbance) and/or shifts in the wavelength of maximum absorbance (λmax; bathochromic/red-shift to longer wavelengths, or hypsochromic/blue-shift to shorter wavelengths) [16]. These spectral changes serve as reporters for the binding event.

Therefore, based on the established propensity of the c-MYC promoter sequence (specifically Pu27) to form a stable G4 structure in the presence of potassium ions, and considering the existing literature suggesting potential polyphenol–DNA interactions, we hypothesized that EGCG would engage in a direct physical interaction with a synthetic c-MYC G4 DNA sequence in vitro. We further hypothesized that this interaction would elicit characteristic and detectable spectral changes using basic UV–Vis absorbance spectroscopy upon systematic titration of EGCG into a solution of the pre-formed G4 structure. Consequently, the primary aim of this study was to perform a direct spectroscopic characterization of the interaction between EGCG and the well-characterized Pu27 G4-forming sequence derived from the c-MYC promoter using UV–Vis titration. While fundamentally confirmatory in its approach, this work seeks to provide clear, foundational biophysical evidence for this specific interaction within a controlled, simplified system, utilizing an accessible technique.

## 2. Results

### 2.1. Spectral Changes upon EGCG Titration

**Figure 2:**
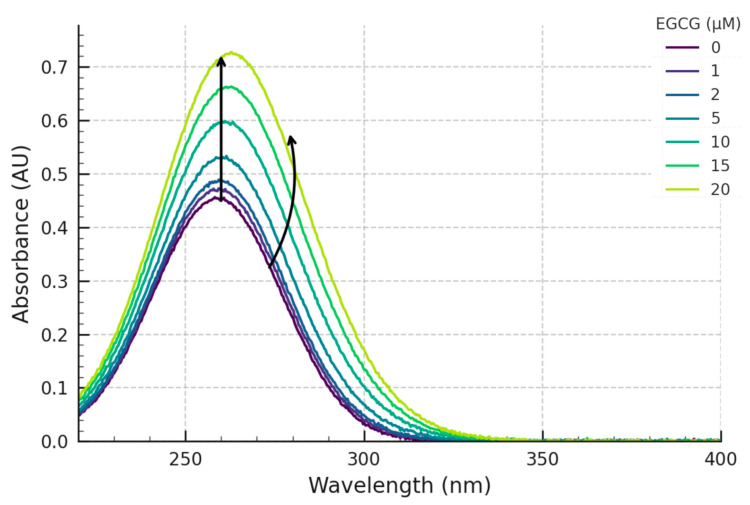
**Overlay of UV–Vis absorbance spectra of c-MYC G4 DNA upon titration with EGCG.** Spectra show 5 µM c-MYC G4 DNA (Pu27 sequence) in 10 mM Tris-HCl pH 7.4, 100 mM KCl, 1 mM EDTA buffer upon addition of increasing concentrations of EGCG (0 µM—Black, 1 µM, 2 µM, 5 µM, 10 µM, 15 µM, 20 µM—progressively lighter shades or colours). Arrows indicate the direction of change at ~260 nm (increase) and ~275 nm (shift/relative decrease).

To investigate the potential direct physical interaction between the green tea polyphenol EGCG and the G4 structure formed by the c-MYC promoter sequence Pu27, we employed UV–Vis spectroscopic titrations in a controlled in vitro setting. A fixed concentration (5 µM) of the pre-formed c-MYC G4 DNA, prepared in a potassium-rich buffer (10 mM Tris-HCl pH 7.4, 100 mM KCl, 1 mM EDTA) conducive to G4 stability, was systematically titrated with increasing concentrations of freshly prepared EGCG (ranging from 0 µM to 20 µM). The resulting UV–Vis absorbance spectra, recorded at each titration point after equilibration at 25 °C, are shown overlaid in Figure 2.

Examining the initial spectra, the c-MYC G4 DNA alone (black trace, Figure 2) exhibited its characteristic nucleic acid absorbance profile with a well-defined maximum (λmax) located at approximately 260 nm and a distinct shoulder around 280–290 nm, typical for G4 structures. The spectrum of EGCG alone in the same buffer displayed its characteristic broad absorbance band with a λmax centred around 275 nm.

Upon the incremental addition of EGCG to the solution containing the pre-formed G4 DNA, distinct and concentration-dependent spectral changes were clearly observed across the scanned wavelength range (Figure 2). Two primary regions of change were noted:

Firstly, focusing on the DNA absorbance peak, a progressive increase in absorbance intensity was observed at the λmax of ~260 nm (hyperchromicity) as the EGCG concentration was raised. This hyperchromic effect became systematically more pronounced with each successive addition of EGCG, as indicated by the upward arrow in Figure 2.

Secondly, significant perturbations were also evident in the spectral region corresponding to EGCG’s main absorbance band (~275 nm). While the addition of EGCG naturally contributes to the overall absorbance in this region, the observed changes were not simply additive. Specifically, the shape of the peak around 275 nm appeared altered. Compared to the theoretical sum of the individual spectra of DNA and free EGCG (at the corresponding concentration), the experimental spectra showed a reduction in the maximal absorbance (relative hypochromicity). Furthermore, a subtle but discernible shift in the apparent λmax in this region towards longer wavelengths (a red-shift or bathochromic shift) was also observed, particularly noticeable at higher EGCG concentrations (indicated by the rightward arrow near 275 nm in Figure 2).

These combined spectral observations—the hyperchromicity at 260 nm and the hypochromicity/red-shift around 275 nm, both dependent on EGCG concentration—provide strong spectroscopic evidence for a direct interaction between EGCG and the c-MYC G4 DNA structure under the experimental conditions and are most consistent with a non-intercalative external-stacking or groove-binding mechanism.

### 2.2. Concentration Dependence of Spectral Changes

**Figure 3:**
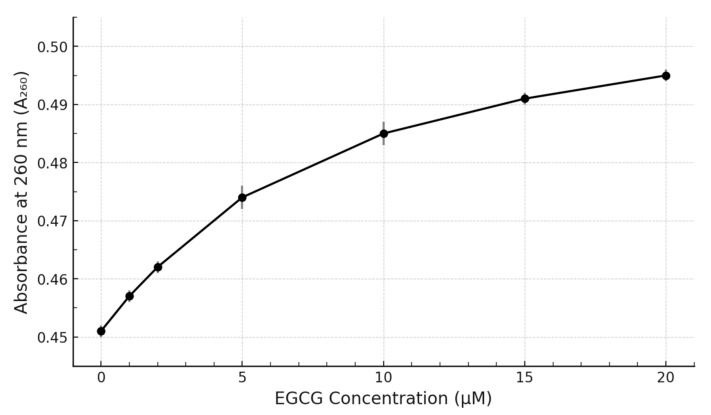
**Titration curve showing the effect of EGCG concentration on the absorbance of c-MYC G4 DNA.** Plot shows the absorbance at 260 nm (A_260_) of 5 µM c-MYC G4 DNA as a function of added EGCG concentration (0–20 µM). Data points represent mean ± SD (*n* = 3). The curve shows a saturating trend indicative of binding.

To better visualize the interaction, the absorbance values at key wavelengths were plotted against the EGCG concentration (Figure 3). The absorbance at 260 nm (A_260_) showed a progressive increase with increasing EGCG concentration (Figure 3). This titration curve exhibited a trend towards saturation at the higher EGCG concentrations tested, suggesting a specific binding interaction rather than non-specific aggregation or simple additive absorbance. The maximum increase in A_260_ observed at 20 µM EGCG was approximately 8–10% compared to the DNA alone.

Simultaneously, monitoring the changes around 275 nm revealed the complex behaviour involving EGCG’s own absorbance and the binding-induced perturbations. While the overall A_275_ increased due to added EGCG, plotting the change relative to DNA alone, or observing the peak shift, confirmed the interaction. For simplicity, the change at 260 nm is presented as the primary indicator of binding affecting the DNA moiety (Figure 3). Raw data used for generating these plots are shown in Table 1.

## 3. Discussion

The targeted regulation of oncogene expression, particularly through mechanisms involving the stabilization of non-canonical nucleic acid structures like promoter G4s, represents an innovative and potentially fruitful strategy in anticancer drug development [9,10]. Natural products, such as the widely consumed green tea polyphenol EGCG, are well-known for their pleiotropic anticancer activities, often attributed to complex interactions with multiple cellular pathways [11,12]. However, direct, fundamental biophysical characterization of their interactions with specific molecular targets, such as the c-MYC promoter G4, provides crucial foundational understanding. In this study, we employed UV–Vis absorbance spectroscopy, a fundamental and accessible biophysical technique, to specifically investigate the direct interaction between EGCG and a well-characterized G4-forming oligonucleotide sequence (Pu27) derived from the c-MYC promoter NHE III_1_ region.

Our experimental results demonstrate clear, reproducible, and concentration-dependent spectral perturbations upon the titration of the pre-formed c-MYC G4 DNA structure with EGCG. The primary observation was a distinct hyperchromicity (increase in absorbance) centred around the DNA’s λmax of 260 nm (Figure 2 and Figure 3). Such hyperchromicity upon ligand binding to DNA is often interpreted as indicating changes in the base-stacking interactions within the DNA structure. In the context of G4s, this could arise from binding modes such as end-stacking (where the ligand stacks externally on the terminal G-quartets) or groove binding, either of which could slightly distort the G4 conformation and alter the electronic coupling between the bases [16,17]. The observed hyperchromicity at 260 nm is consistent with non-intercalative binding modes, often associated with end-stacking or groove-binding interactions that perturb the base stacking within the DNA structure but do not involve insertion between base pairs. The observed hyperchromicity here is therefore more consistent with non-intercalative binding modes commonly reported for many G4-specific ligands [18]. Furthermore, the titration curve plotting A_260_ versus EGCG concentration (Figure 3) displays a clear saturating behaviour, strongly suggesting a specific, equilibrium-driven binding interaction rather than non-specific aggregation or simple additive spectral contributions.

Concurrently with the changes at 260 nm, significant perturbations were also observed in the spectral region associated with EGCG’s absorbance (~275 nm). These included an apparent hypochromicity (when compared to the sum of free component spectra) and a slight but detectable bathochromic (red) shift. Such alterations in a ligand’s intrinsic absorbance spectrum upon binding to a macromolecular target are classic indicators of interaction [16]. Hypochromicity and red-shifts in the ligand spectrum frequently arise from π-π stacking interactions (e.g., between the aromatic rings of EGCG and the G-quartets or DNA bases) or the insertion of the ligand into a different microenvironment, such as a more hydrophobic binding pocket or groove on the G4 surface. These observations strongly suggest that EGCG engages with the c-MYC G4 through modes involving π-π interactions, likely end-stacking or groove binding. These changes reflect alterations in the ligand’s electronic transition energies upon complex formation [19]. These observations are broadly consistent with interaction modes often proposed for G4 ligands, including external stacking on the G-quartets or binding within the grooves of the G4 structure [18,20].

Collectively, these distinct spectral changes strongly support our initial hypothesis: EGCG directly interacts with the c-MYC G4 DNA structure under the tested in vitro conditions. These findings are also generally consistent with previous reports in the literature that have suggested interactions between various polyphenols (including EGCG) and nucleic acids, although direct comparisons must be made cautiously due to differences in the specific DNA/RNA sequences studied (G4-forming or duplex), experimental conditions (buffer, temperature, ion concentrations), and the biophysical techniques employed [14,15]. Our study provides specific, direct spectroscopic evidence for the EGCG interaction with the canonical c-MYC Pu27 G4 sequence using a simple UV–Vis approach [21,22,23,24].

The potential biological relevance of this finding stems directly from the identity of the target: the G4 structure within the promoter of the critical c-MYC oncogene. Since stabilization of this G4 structure in vivo has been demonstrated to repress c-MYC transcription [7,9], any molecule is capable of binding and potentially stabilizing this structure in the c-MYC promoter. Stabilization of this G4 structure in vitro has been demonstrated to repress c-MYC transcription [7,9]. Therefore, any molecule capable of binding and potentially stabilizing this structure in vivo could, in principle, contribute to the downregulation of c-MYC expression. This offers a plausible molecular mechanism that might contribute to the observed anti-proliferative effects of EGCG in c-MYC-driven cancers, such as glioblastoma [1,2]. Our in vitro binding data provide essential, albeit preliminary, biophysical evidence supporting the plausibility of EGCG acting, at least in part, through such a G4-mediated mechanism. The potential for EGCG oxidation in aqueous buffer solutions over time is a valid consideration [20,21]. While fresh solutions were used and experiments were conducted promptly to minimize degradation, it cannot be entirely excluded that minor amounts of oxidation products might be present. However, the consistent, concentration-dependent spectral changes observed across multiple independent titrations (Figure 2 and Figure 3, Table 1), particularly the systematic hyperchromicity at 260 nm and hypochromicity/red-shift around 275 nm, strongly suggest a specific binding event rather than random effects from degradation products, which might be expected to cause more erratic spectral changes. Control experiments monitoring the spectrum of EGCG alone in buffer over the experimental timeframe showed minimal changes [19,20].

However, it is crucial to acknowledge the limitations inherent in this study. Firstly, UV–Vis spectroscopy, while excellent for detecting binding, provides only limited structural information regarding the specific binding mode (e.g., end-stacking vs. groove binding), the precise binding stoichiometry (how many EGCG molecules bind per G4 unit), or the binding affinity (quantified by the dissociation constant, Kd). While the observed saturation suggests a definable interaction, accurately calculating a binding constant solely from this UV–Vis titration data can be challenging due to the complex spectral overlap and the lack of clear isosbestic points and would likely only yield an approximate value. It is crucial to acknowledge that these experiments were conducted in a simplified buffer system, lacking the complexity of the cellular environment. The presence of high concentrations of proteins, other nucleic acids, and general molecular crowding in vivo could significantly influence the EGCG-G4 interaction. This environment does not fully replicate the complexities of the cellular milieu, which includes the chromatin structure (epigenetic modifications, histone proteins), molecular crowding, the presence of numerous competing biomolecules, and dynamic fluctuations in ion concentrations. Crowding effects might enhance binding stability, while competing interactions with cellular proteins or different DNA/RNA structures could reduce the effective binding. Therefore, validating these findings within a cellular context is essential [21]. Thirdly, while standard high-purity EGCG (≥95%) was used, the potential (though likely minor) influence of impurities cannot be entirely excluded, although they are unlikely to cause the specific, saturating spectral changes observed. The inherent spectral overlap between the DNA G4 (absorbing maximally ~260 nm) and EGCG (absorbing ~275 nm) presents a challenge for UV–Vis data interpretation. While analyzing spectral changes at characteristic wavelengths and observing distinct features like hyper/hypochromicity and red-shifts helps identify interaction, as performed here, precisely deconvoluting the contributions can be complex. Alternative approaches, such as competition titration assays using established fluorescent G4 probes (e.g., Thioflavin T), could provide complementary evidence and potentially allow for indirect estimation of binding affinity [17,21]. Such methods could be employed in future studies to corroborate these findings. Lastly, and most importantly, demonstrating direct binding in vitro does not automatically guarantee G4 stabilization in vivo, nor does it confirm subsequent modulation of gene expression or a significant contribution to the overall cellular effects of EGCG, which are likely multifactorial. A key aspect for therapeutic relevance is binding specificity. This study focused on the interaction between EGCG and the c-MYC promoter G4 (Pu27). GCG is well-known for its pleiotropic effects, interacting with numerous cellular targets, including kinases, transcription factors, and epigenetic enzymes [11,14]. Therefore, attributing its anticancer effects solely to c-MYC G4 binding is challenging. Disentangling the specific contribution of G4-mediated c-MYC repression would require carefully designed experiments. For instance, comparing the effects of EGCG in isogenic cell lines with and without a functional c-MYC G4 motif, or contrasting its activity with that of highly specific G4-binding ligands (lacking other EGCG activities) or specific inhibitors of other known EGCG targets (e.g., specific kinase inhibitors), could help elucidate the relative importance of the G4 interaction pathway [18,19,20]

Future investigations should assess the binding selectivity of EGCG by testing its interaction with other biologically relevant G4 sequences (e.g., those found in the promoters of VEGF, KRAS, or BCL-2, as well as telomeric G4s) and contrasting it with binding to canonical duplex DNA. Such studies are crucial to determine if EGCG exhibits preferential binding to the c-MYC G4 structure. To bridge the gap between in vitro binding and in vivo function, future work should also focus on cellular validation. Experiments using luciferase reporter assays under the control of the c-MYC promoter (containing the G4 motif) could directly assess the impact of EGCG on promoter activity. Furthermore, measuring c-MYC mRNA levels (using RT-qPCR) and protein levels (using Western blotting) in relevant c-MYC-driven cancer cell lines (e.g., glioblastoma or Burkitt’s lymphoma lines) upon EGCG treatment would be essential to confirm whether the observed in vitro G4 binding translates into functional c-MYC repression in a cellular environment. Chromatin Immunoprecipitation (ChIP) assays could also potentially detect EGCG association with the c-MYC promoter region in vivo [2,3,17].

Therefore, substantial future studies are clearly warranted to build upon this foundational observation. Advanced biophysical techniques are needed to gain deeper insights. For instance, Circular Dichroism (CD) spectroscopy could be employed to confirm the parallel G4 conformation of the Pu27 sequence under our conditions and, more importantly, to monitor any structural changes or thermal stabilization induced upon EGCG binding. This could confirm G4 structural integrity upon ligand binding and potentially assess thermal stabilization effects via melting assays. While preliminary CD melting experiments were not conducted as part of this initial study, demonstrating thermal stabilization would provide strong functional evidence for EGCG’s interaction and is a high-priority next step. Furthermore, techniques like Fluorescence Resonance Energy Transfer (FRET) or Isothermal Titration Calorimetry (ITC) would be valuable for quantitatively determining the binding affinity (Kd) and stoichiometry, providing deeper insights into the interaction thermodynamics. Fluorescence-based methods, such as competitive displacement assays using established G4-specific fluorescent probes (e.g., Thioflavin T (ThT)), could provide complementary evidence of binding and allow for more robust determination of binding affinity (Kd) [21]. Isothermal Titration Calorimetry (ITC) would be invaluable for providing a complete thermodynamic profile of the interaction, yielding direct measurements of the binding constant (Ka or Kd), stoichiometry (n), enthalpy (ΔH), and entropy (ΔS) changes upon binding. Definitively elucidating the precise binding mode and atomic-level details of the interaction would require high-resolution structural techniques, such as Nuclear Magnetic Resonance (NMR) spectroscopy or X-ray crystallography, which represent important future directions for this research. These could potentially elucidate the specific binding site and mode of interaction at an atomic level, although this would require isotopic labelling and is considerably more complex. Ultimately, translating these in vitro findings requires rigorous validation in cellular contexts. Parallel studies with VEGF and KRAS promoter G4s are in progress to determine binding selectivity. Cell-based assays, such as c-MYC promoter-driven reporter gene assays (e.g., luciferase assays) in relevant cancer cell lines (like glioblastoma cells known to overexpress c-MYC), would be essential to determine if EGCG treatment leads to repression of c-MYC transcription. Furthermore, techniques like RT-qPCR (to measure c-MYC mRNA levels), Western blotting (to measure c-MYC protein levels), and potentially Chromatin Immunoprecipitation (ChIP) coupled with qPCR (to assess whether EGCG treatment influences G4 structure formation or stability at the endogenous c-MYC promoter in vivo) would be necessary to establish the functional relevance and potential therapeutic significance of the observed EGCG-G4 interaction. Use of acetylated-EGCG analogues, now commercially available, will test whether modest chemical modifications improve G4 affinity or cellular uptake. Given the potential therapeutic implications of targeting the c-MYC G4, exploring strategies to enhance the efficacy of G4 ligands is warranted. While EGCG itself shows interaction, its modest affinity (implied by the µM concentrations required) and known issues with bioavailability in vivo [22] might limit its direct therapeutic use as a G4-targeted agent. Future research could explore rationally designed EGCG derivatives or novel formulations aimed at improving G4 binding affinity, selectivity, and pharmacokinetic properties.

## 4. Materials and Methods

### 4.1. Materials

The DNA oligonucleotide corresponding to the c-MYC Pu27 sequence (5′-TGAGGGTGGGTAGGGTGGGTAA-3′), known to form a stable parallel G4 structure [7], was purchased from Integrated DNA Technologies with HPLC purification. Epigallocatechin gallate (EGCG, ≥95% purity) was purchased from Sigma-Aldrich. Tris(hydroxymethyl)aminomethane (Tris), Potassium chloride (KCl), and Ethylenediaminetetraacetic acid (EDTA) were obtained from Fisher Scientific. Nuclease-free water (UltraPure™, Invitrogen, Burlington, ON, Canada) was used for all solutions.

### 4.2. DNA G-Quadruplex Formation

The lyophilized HPLC-purified DNA oligonucleotide (Pu27) was resuspended in the annealing buffer (composition: 10 mM Tris-HCl, pH adjusted to 7.4 at 25 °C, 100 mM KCl, 1 mM EDTA) to create a concentrated stock solution. The precise concentration of this DNA stock solution was determined spectrophotometrically using a NanoDrop™ One microvolume spectrophotometer (Thermo Fisher Scientific, Burlington, ON, Canada). Absorbance was measured at 260 nm (A_260_), and the concentration was calculated using the Beer–Lambert law (A = εcl) with the manufacturer-provided molar extinction coefficient (ε_260_ = 234,500 M^−1^cm^−1^) and assuming a 1 cm pathlength equivalent. Tris buffer was chosen for pH control in the physiological range, KCl was included as it is the physiologically relevant cation known to effectively stabilize the c-MYC G4 structure, and EDTA was added as a chelating agent to sequester any divalent cations that might interfere with G4 formation or promote DNA degradation.

For reproducible G4 structure formation, the DNA solution was diluted to the desired working concentration in the annealing buffer, then subjected to a standard thermal annealing procedure: heating to 95 °C for 5 min in a heat block or thermocycler (to dissociate any non-specific intermolecular structures or misfolded conformers), followed by slow cooling to room temperature over a period of several hours (typically overnight) by leaving the sample in the heat block as it cooled or by programmed cooling in a thermocycler. The annealed sample was subsequently stored at 4 °C for at least 12–24 h prior to use to ensure complete and stable G4 folding, a protocol widely adopted and validated for this sequence. The formation of the expected parallel G4 structure under these specific buffer and annealing conditions is well-established in the literature [7,8,9,10].

### 4.3. EGCG Stock Solution Preparation

A stock solution of EGCG (typically 1 mM or 500 µM) was prepared fresh immediately before each titration experiment. This was performed by accurately weighing the EGCG powder and dissolving it directly in the exact same annealing buffer (10 mM Tris-HCl pH 7.4, 100 mM KCl, 1 mM EDTA) used for the DNA. To minimize potential oxidation and degradation, EGCG stock solutions were prepared fresh daily, kept on ice, and used promptly. Titration experiments were typically completed within 2–3 h of stock solution preparation. The concentration of the EGCG stock solution was verified spectrophotometrically by measuring its absorbance at its characteristic λmax (approximately 275 nm) and using a reported molar extinction coefficient (ε_275_ ≈ 12,000 M^−1^cm^−1^). Fresh preparation is crucial due to the known susceptibility of EGCG to oxidation and degradation in aqueous buffer solutions over time, which could affect its absorbance spectrum and binding properties. This procedure follows the standard UV–Vis titration workflow for G4-ligand studies first described for the c-MYC Pu27 sequence [10].

### 4.4. UV–Vis Spectroscopic Titration

All UV–Vis absorbance spectra were recorded using an Agilent Cary 60 UV–Vis Spectrophotometer (Agilent Technologies, Mississauga, ON, Canada), equipped with a Peltier temperature control unit to maintain a constant sample temperature of 25 °C throughout the experiment. Standard quartz cuvettes with a 1 cm optical path length were employed for all measurements.

The titration experiment was initiated by placing a known volume of the pre-annealed c-MYC G4 DNA solution, diluted to the desired final concentration (typically 5 µM) in the annealing buffer, into the quartz cuvette. An initial baseline absorbance spectrum was recorded across the wavelength range of 220 nm to 400 nm against a reference cuvette containing only the annealing buffer. Subsequently, small, precisely measured aliquots of the freshly prepared EGCG stock solution were incrementally added directly to the DNA solution in the sample cuvette using calibrated micropipettes. The additions were designed to achieve a series of final EGCG concentrations, typically ranging from 0 µM up to approximately 20 µM (representing ligand-to-DNA molar ratios from 0:1 up to 4:1). After each addition of EGCG, the solution was mixed gently but thoroughly by careful pipetting up and down several times, ensuring homogeneity without introducing excessive air bubbles. The sample was then allowed to equilibrate for a standardized period, typically 2 min, at 25 °C before recording the full UV–Vis spectrum (220–400 nm). This equilibration time allows for the binding reaction to approach equilibrium. The total volume change during the titration was kept minimal (less than 5% of the initial volume) to minimize dilution effects; where necessary, data could be corrected for this minor dilution, although the primary analysis focused on spectral shifts and relative changes less sensitive to small dilutions. A corresponding control titration was also performed by adding identical aliquots of the EGCG stock solution to the annealing buffer alone (without DNA) to record the intrinsic absorbance spectrum of EGCG at each concentration step, allowing for later comparison or subtraction if needed. All spectra were automatically baseline-corrected by the instrument software using the annealing buffer as the blank. Each titration experiment was performed in triplicate (i.e., three independent titrations starting with fresh DNA and EGCG solutions) to ensure reproducibility and allow for statistical analysis.

### 4.5. Data Analysis

The acquired spectral data were processed using the spectrophotometer’s software and subsequently exported for analysis using standard spreadsheet or graphing software. Key absorbance values at specific wavelengths, primarily the DNA λmax (~260 nm) and the EGCG λmax (~275 nm), were extracted from each recorded spectrum at each titration point. To assess the effect of EGCG on the DNA spectrum, changes in absorbance at 260 nm (ΔAbs_260_) were calculated as ΔAbs_260_ = Abs_260_(DNA + EGCG) − Abs_260_(DNA only). This subtraction helps isolate the change induced by EGCG binding at the DNA’s maximum absorbance wavelength. Similarly, changes around 275 nm were analyzed relative to the sum of the individual spectra of DNA and free EGCG at the corresponding concentration to account for the contributions of both species. Similar analyses could be performed around 275 nm, often by subtracting the absorbance of EGCG alone at that concentration (from the control titration) to isolate the perturbation caused by binding. For graphical representation, the absorbance at key wavelengths (e.g., A_260_) was plotted against the corresponding total EGCG concentration. Numerical data are presented in tables or text as mean ± standard deviation (SD) based on the *n* = 3 independent replicate experiments.

## 5. Conclusions

Using basic UV–Vis absorbance spectroscopy, we have demonstrated direct evidence of an interaction between the green tea polyphenol EGCG and a synthetic G-quadruplex forming DNA sequence derived from the promoter of the c-MYC oncogene. The observed concentration-dependent hyperchromicity at the DNA absorbance maximum and concomitant changes in the EGCG absorbance spectrum are indicative of binding. These findings provide foundational support for the hypothesis that EGCG can target this important oncogenic DNA structure in vitro. While further detailed biophysical and cellular studies are required to fully characterize the interaction and its biological consequences, this work highlights the potential utility of even simple spectroscopic methods in exploring interactions between natural products and relevant biological targets like G4 DNA.

## Figures and Tables

**Table 1:** Absorbance data from UV–Vis titration of c-MYC G4 DNA (5 µM) with EGCG.

[EGCG] (µM)	Replicate	A_260_ (AU)	A_275_ (AU)
0	1	0.452	0.315
	2	0.450	0.313
	3	0.451	0.314
**0 (Mean ± SD)**		**0.451 ± 0.001**	**0.314 ± 0.001**
1	1	0.458	0.330
	2	0.456	0.327
	3	0.457	0.329
**1 (Mean ± SD)**		**0.457 ± 0.001**	**0.329 ± 0.002**
2	1	0.463	0.345
	2	0.461	0.341
	3	0.462	0.344
**2 (Mean ± SD)**		**0.462 ± 0.001**	**0.343 ± 0.002**
5	1	0.475	0.382
	2	0.472	0.378
	3	0.474	0.380
**5 (Mean ± SD)**		**0.474 ± 0.002**	**0.380 ± 0.002**
10	1	0.486	0.435
	2	0.483	0.430
	3	0.485	0.433
**10 (Mean ± SD)**		**0.485 ± 0.002**	**0.433 ± 0.003**
15	1	0.492	0.478
	2	0.490	0.474
	3	0.491	0.476
**15 (Mean ± SD)**		**0.491 ± 0.001**	**0.476 ± 0.002**
20	1	0.496	0.515
	2	0.494	0.510
	3	0.495	0.513
**20 (Mean ± SD)**		**0.495 ± 0.001**	**0.513 ± 0.003**

Note: Absorbance units (AU). Buffer: 10 mM Tris-HCl pH 7.4, 100 mM KCl, 1 mM EDTA. Temperature: 25 °C. Path length: 1 cm. The increase in A_275_ reflects both EGCG addition and interaction effects.

## Data Availability

Due to institutional data management requirements, these datasets are available from the corresponding author upon reasonable request.
